# Epigenetics in Schizophrenia: A Pilot Study of Global DNA Methylation in Different Brain Regions Associated with Higher Cognitive Functions

**DOI:** 10.3389/fpsyg.2016.01496

**Published:** 2016-09-30

**Authors:** Raúl Alelú-Paz, Francisco J. Carmona, José V. Sanchez-Mut, Ariel Cariaga-Martínez, Ana González-Corpas, Nadia Ashour, Maria J. Orea, Ana Escanilla, Alfonso Monje, Carmen Guerrero Márquez, Jerónimo Saiz-Ruiz, Manel Esteller, Santiago Ropero

**Affiliations:** ^1^Biochemistry and Molecular Biology Unit, Department of Systems Biology, School of Medicine, University of AlcaláMadrid, Spain; ^2^Laboratory for Neuroscience of Mental Disorders Elena Pessino, Department of Medicine and Medical Specialties, School of Medicine, University of AlcaláMadrid, Spain; ^3^Department of Psychiatry, CIBERSAM, IRYCIS, Hospital Ramón y CajalMadrid, Spain; ^4^Cancer Epigenetics and Biology Program, Bellvitge Biomedical Research Institute, L'Hospitalet de LlobregatBarcelona, Spain; ^5^Neurological Brain Bank, Parc Sanitari Sant Joan de DéuBarcelona, Spain; ^6^Biobank-Brain Bank of University Hospital Alcorcón FoundationMadrid, Spain; ^7^Institució Catalana de Recerca i Estudis AvançatsBarcelona, Spain; ^8^Department of Physiological Sciences II, School of Medicine, University of BarcelonaBarcelona, Spain

**Keywords:** psychology, cognition, executive function, memory, epigenetics, DNA methylation, schizophrenia, human brain

## Abstract

Attempts to discover genes that are involved in the pathogenesis of major psychiatric disorders have been frustrating and often fruitless. Concern is building about the need to understand the complex ways in which nature and nurture interact to produce mental illness. We analyze the epigenome in several brain regions from schizophrenic patients with severe cognitive impairment using high-resolution (450K) DNA methylation array. We identified 139 differentially methylated CpG sites included in known and novel candidate genes sequences as well as in and intergenic sequences which functions remain unknown. We found that altered DNA methylation is not restricted to a particular region, but includes others such as CpG shelves and gene bodies, indicating the presence of different DNA methylation signatures depending on the brain area analyzed. Our findings suggest that epimutations are not relatables between different tissues or even between tissues' regions, highlighting the need to adequately study brain samples to obtain reliable data concerning the epigenetics of schizophrenia.

## Introduction

Schizophrenia is one of the most complex and enigmatic of all psychiatric disorders, being characterized by the heterogeneous presence of positive, negative, and cognitive symptoms that affect all aspects of mental activity. Twin, family, and adoption studies support the idea that schizophrenia has a strong genetic component, with an estimated heritability as high as ~80% (Owen et al., 2002; Brennand et al., 2011). However, genetics alone cannot explain its incidence. In this way, the absence of consistently reproducible molecular defects, and the evidence for long-lasting changes in gene expression patterns after environmental exposures suggest that epigenetic mechanisms play a crucial role in psychiatric diseases, since it is well-established that environmental exposures can modify DNA methylation patterns (Feil and Fraga, 2011).

However, our knowledge about the effect of epigenetic modifications on the development of this disease is in its infancy. To date, five epigenome-wide association studies (EWAS) have been published that analyze the DNA methylation profile of schizophrenic patients. Four of these were performed in peripheral blood samples and one used the frontal cortex of schizophrenic patients (Mill et al., 2008; Dempster et al., 2011; Kinoshita et al., 2013; Nishioka et al., 2013; Liu et al., 2014). These EWAS are comparable to highly successful genome-wide association studies but gave mixed results (Rakyan et al., 2011) describing different altered DNA methylation patterns. This prompted us to pursue this line of study in an attempt to obtain independent replications of previously reported epigenetic alterations, but including new clinical and anatomical variables.

Anatomical structures in normal brain are responsible for maintaining adequate cognitive functions. For instance, prefrontal cortex plays a critical role in supporting emotional behavior as well as sociomoral processing and executive functions (Barrasso-Catanzaro and Eslinger, 2016). Other structures, as hippocampus were related to key functions in spatial (O'Keefe and Dostrovsky, 1971) and episodic memory (Tulving, 2002), and its underlying molecular pathways are still under research (Mitsumori, 2008). Even more, anatomical changes of these structures are related to mental disorders. For instance, schizophrenic patients showed reduced levels of gray matter volume in anterior cingulate cortex (ACC; Yamasue et al., 2004) and the connectivity of this structure with other brain regions could also play a role in first-episode schizophrenia development (Ohtani et al., 2015) and might be related to negative symptoms in schizophrenia (Ohtani et al., 2014).

Given that the spectrum of cognitive defects and symptoms in schizophrenic patients might be associated with defects in specific brain structures, our study aimed to compare the methylation profile of the dorsolateral prefrontal cortex (DLPFC), hippocampus and ACC of healthy controls and of patients with a diagnosis of schizophrenia with severe cognitive deficits in the executive function, memory and with patterns of positive and negative symptoms, respectively, by using Illumina's 450K DNA methylation microarray (Infinium HumanMethylation450 BeadChip). This microarray encompass 485,764 CpGs located along the human genome, and exceeds the number of CpGs covered by Illumina's previous EWAS, in which the analysis was limited to the CpG sites in CpG islands in the gene promoter region. Although, we previously have described the methylation status of the major neurotransmitter systems associated with the pathophysiology of schizophrenia (Alelu-Paz et al., 2015) to our knowledge, the present study is the first to perform this genome-wide methylation array in several brain regions from schizophrenic patients with severe cognitive impairment, and so is a unique epigenetic analysis of the normal and schizophrenic human brain.

## Materials and methods

### Neuropsychological test batteries

We administered a comprehensive battery of neurocognitive tests to all patients in random order. The battery included the Buschke Memory Impairment Screen (MIS; Buschke et al., 1999), the Frontal Assessment Battery (FAB; Rodriguez del Alamo et al., 2003), the Mini Mental State Examination (MMSE; Lobo et al., 1999), and the Positive and Negative Syndrome Scale (PANSS; Kay et al., 1990), all of which have been validated for the Spanish population. The MIS is a quick (3–4 min), four-item, delayed free, and cued recall test of memory impairment. This test uses controlled learning to ensure attention, induce specific semantic processing and optimize encoding specificity to improve detection of cognitive impairment (in this test, scores from 0 to 3 represent high cognitive impairment and 4–5 represent mild cognitive impairment. Mean age ± *SD* = 6.1 ± 2). The FAB is a short cognitive and behavioral six subtests devised for screening of a global executive dysfunction (scores from 0 to 10 represent high cognitive impairment and from 10 to 15 represent mild cognitive impairment. Mean age ± *SD* = 10.3 ± 4.7). The MMSE is a short, standardized form devised for screening the cognitive mental state (scores from 10 to 20 represent mild cognitive impairment and <10 represent high cognitive impairment. Mean age ± *SD* = 25.4 ± 3.4). Finally, PANSS was conceived as an operationalized, drug-sensitive instrument that provides balanced representation of positive and negative symptoms and gauges their relationship to one another and to global psychopathology.

We assessed each patient's cognitive status at least twice over a period of 24 months prior to the death.

### Samples

We selected those deceased patients who had presented a severe impairment in executive function, memory, or positive/negative symptoms in the neuropsychological evaluations described above. The samples were obtained using the Atlas of the Human Brain (Mai et al., 2004), in order to obtain the same regions in each subject. We included frozen post-mortem brain samples from the DLPFC, hippocampus, and ACC of subjects diagnosed with schizophrenia by DSM-IV-TR criteria and of healthy controls. We obtained the samples after the corresponding written consents were given by the healthy subjects, the patients or their relatives. They were included in the study after obtaining the approval of the corresponding ethical committees of the different institutions (Department of Pathology of the Hospital Ramón y Cajal, Madrid, Spain; the Brain Bank at the University Hospital Alcorcón Foundation, Madrid, Spain; and the Neurological Brain Bank of Sant Joan de Déu-Serveis de Salut Mental, Barcelona, Spain). Table 1S summarizes the characteristics (post-mortem interval, age at death, psychiatric diagnosis, and sex) of the frozen tissue sample (see Supplementary Material). We received sections in dry ice and maintained them at −80°C until use. In the study, we included six schizophrenic samples of DLPFC with a severe executive function deficit, six schizophrenic samples of hippocampus with severe memory impairment and seven schizophrenic samples of ACC with positive and negative symptoms (two and five samples, respectively). The control group consisted of three healthy DLPFC, hippocampus, and ACC samples.

The criteria for including cases in control samples included a post-mortem delay no longer than 24 h and no clinical or pathological evidence of neurological or psychiatric disease. For pathological cases the inclusion criteria included a post-mortem delay no longer than 24 h, a diagnosis of schizophrenia (DSM-IV-TR criteria for residual subtype) and an exhaustive neuropsychological evaluation.

All subjects are high-rate smokers and polymedicated patients and, therefore, it is not possible to analyze the influence of specific drug on the DNA methylation patterns.

### DNA extraction

DNA isolation was performed following the protocol established by Alelu-Paz et al. (2015) for human brain samples.

### Identification of differentially methylated CpGs

After bisulfite conversion of 500 ng of each sample, we used 4 μl of bisulfite-converted DNA to hybridize on the Infinium HumanMethylation450 Beadchip, following the Illumina Infinium HD Methylation protocol. Detailed information about the contents of this array is available in the Infinium HumanMethylation450 Beadchip user guide and data sheet. Two previous papers described the technical schemes, the accuracy and the high reproducibility of this array (Sandoval et al., 2011; Kinoshita et al., 2013). To analyze the DNA methylation data we used the methylation analysis module available within the BeadStudio program (Illumina, Inc.).

To calculate the DNA methylation status of the CpG sites, we selected the β-value, which represents the signal ratio from a methylated probe relative to the sum of the methylated and unmethylated probes, and takes any value between 0 (unmethylated) and 1 (completely methylated). To avoid possible sources of technical bias that might influence the results, we excluded probes located on the X chromosome and every β value that had a threshold detection value of *p* > 0.01. GenomeStudio normalizes data using internal controls included in the HumanMethylation 450 BeadChip, and also normalizes data with respect to internal background probes. To avoid any potential false positive results due to the restricted number of samples, we have performed an accurate statistical analysis, considering CpG methylation differences above a cut-off increment of 0.2 (20% of methylation) in the β-value unless specifically indicated otherwise.

### Gene functional classification and protein-protein interaction networks

To identify functions associated with the genes analyzed, we performed gene ontology (GO) analysis using the *Database for Annotation, Visualization, and Integrated Discovery* program (DAVID v6.7; http://david.abcc.ncifcrf.gov), which is able to extract biological features and meaning associated with gene lists, and which has most commonly been employed in genome-wide or near-genome-wide studies (Dennis et al., 2003). We used the STRING database v9.05 (http://string-db.org/; Jensen et al., 2009) to identify all the direct and indirect interactions between the proteins coded by the genes obtained in our analysis that operate in coordination with others to enable different biological processes. Transcription factor binding sites predictory analysis were performed by using tools from the UCSC Genome Browser (https://genome.ucsc.edu/).

## Results

**Figure 1:**
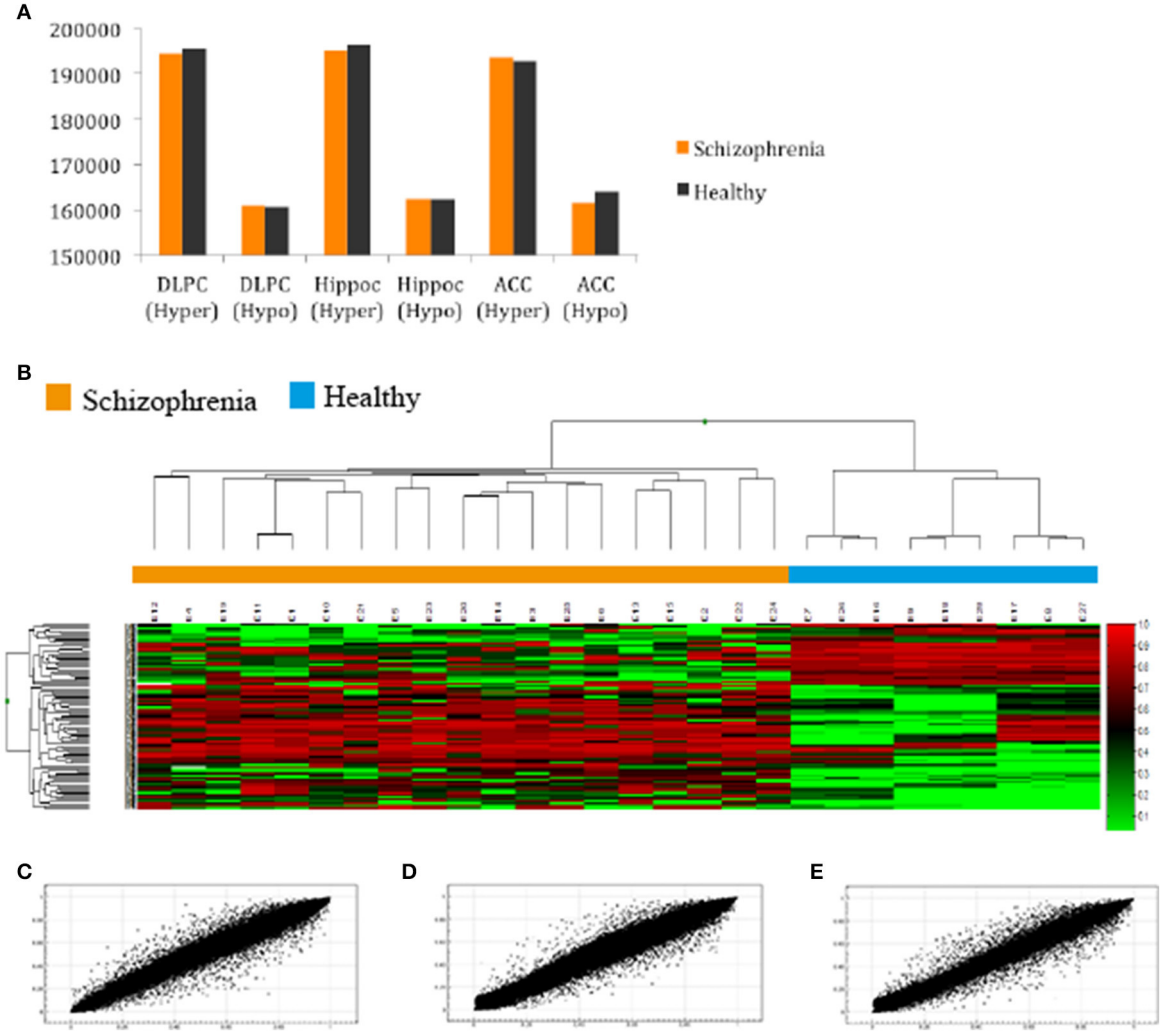
**(A)** Total number of hyper and hypomethylated CpG sites both in schizophrenic and healthy samples; **(B)** Hierarchical cluster analysis of 301 probes in the total samples included in our study with a 0.20-fold change in β-values as the cut-off (*SD* < 30%; Green, samples with lowest methylation level; Red, samples with highest methylation level). Cluster analysis discriminated between schizophrenic patients (orange box) and control samples (blue box); **(C,D,E)** Representative example of the scatter plot for CpG methylation values (AVGβ) between healthy and schizophrenic samples in the **(A)** DLPC (*R*^2^ = 0.9945); **(B)** Hippocampus (*R*^2^ = 0.9949); and **(C)** ACC (*R*^2^ = 0.9931).

After filtering the data as described in the Section Materials and Methods, 288 probes (0.06%) were discarded and 485,476 probes (99.94%) were used for further analysis. First, we compared the total number of methylated (β ≥ 0.75) and unmethylated (β ≤ 0.20) CpG sites in schizophrenic and healthy samples. In general terms, intragroup analysis showed that, in all brain structures, there were more methylated than unmethylated CpGs in the schizophrenic and normal brain (*Z*-test; *p* < 0.01). More interestingly, in the intergroup analysis we found significantly more methylated CpGs in schizophrenic DLPFC and hippocampus than in the corresponding healthy brain structures, while more unmethylated probes were found in the schizophrenic ACC than in the healthy ACC (*Z*-test; *p* < 0.01; Figure 1A and Table 2S). The corresponding global hierarchical clustering analysis of the methylation state of probes with Δβ-values of >0.2 (*n* = 301; *p* < 0.01, *SD* < 30%) shows that differentially methylated CpG positions distinguished schizophrenic from healthy samples (Figure 1B).

**Figure 2:**
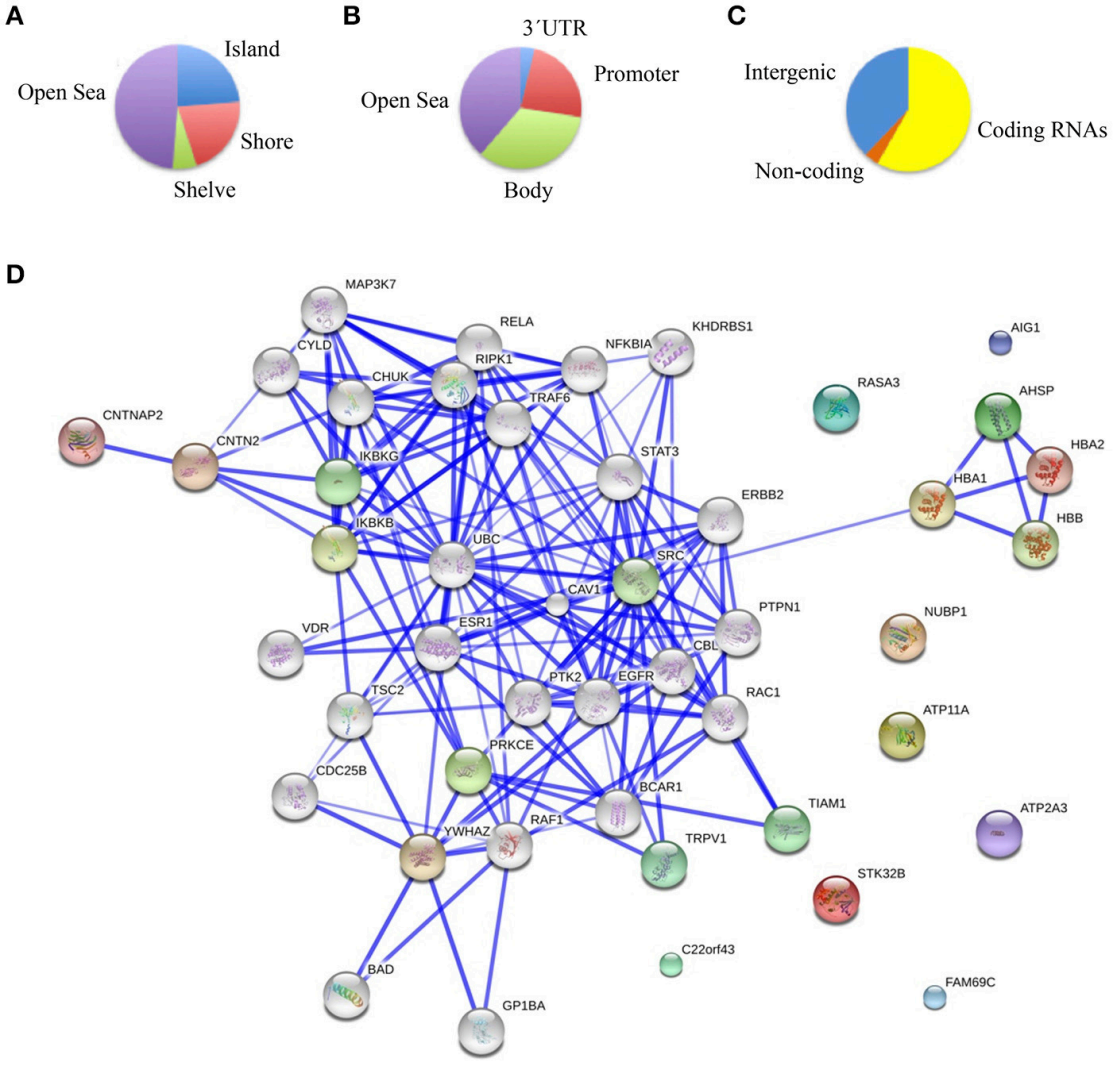
**(A)** Functional genomic distribution, **(B)** CpG content and neighborhood context, and **(C)** associated RNA transcripts of the 66 differentially methylated CpGs between schizophrenic and healthy samples in the DLPC; **(D)** Network analysis using STRING (V9.05) identified different protein-protein associations. Stronger associations are represented by thicker lines.

Scatterplots comparing the average DNA methylation levels of the two groups indicate that although the correlation was high, the methylation profile of a number of CpGs sites differed between schizophrenic and healthy DLPFC (Pearson, *R*^2^ = 0.993), hippocampus (*R*^2^ = 0.994), and ACC (*R*^2^ = 0.993; Figures 1C–E, respectively). Scatterplots comparing brain areas (DLPFC vs. Hippocampus, DLPFC vs. ACC, and Hippocampus vs. ACC) are included in the Supplementary Material (Figures 1S, 2S).

The main objective of this study was to determine the DNA methylation changes associated with the spectrum of cognitive defects and symptoms related to chronic schizophrenia by comparing the DNA methylation pattern of the brain structures associated with specific cognitive domains, such as DLPFC with executive function, hippocampus with memory, and ACC with positive and negative symptoms. Therefore, we focused on the differences in the methylation patterns in each of these structures. To this end we considered as differentially methylated probes those with a Δβ > 0.2 between groups (*p* < 0.01, *SD* < 20%). Bonferroni correction for multiple testing was applied at the <10^−7^ level. When we compared schizophrenic and healthy DLPFCs, we found 66 differentially methylated probes corresponding to 37 genes (Table 3S) with a similar proportion of hypermethylated (35/66) and hypomethylated (31/66) CpG sites in the schizophrenic compared with control samples. Table 1 summarizes the 10 most differentially methylated probes (*p* < 0.01; β > 0.2) in the three brain areas analyzed between schizophrenic and healthy samples. From the CpG content and neighborhood context of the 66 differentially methylated CpGs, 24.05% were in CpG islands, 21.5% in CpG shores, 6.3% in CpG shelves, and 48.1% were outside coding genes or known transcription regulatory elements (intergenic-open sea; Figure 2A). From the functional genome distribution standpoint, 24% of CpGs were located in proximal promoters (defined as the sum of CpG sites located within 200 or 1500 bp upstream of the described transcription start site, 5′UTR and exon 1), 3.7% CpG sites were located in 3′UTR and, finally, 34.1 and 37.9% CpGs corresponded to gene body and intergenic-open sea sequences, respectively (Figure 2B). According to their associated RNA transcripts, 58.2% corresponded to classic coding messenger RNA genes, 3.7% were linked to non-coding RNAs, and 37.97% of CpG sites had no associated annotated transcripts (Figure 2C). It is important to highlight that we found in this brain area fewer probes associated with promoter regions and coding transcripts included in the 450K (more than 15% points; Sandoval et al., 2011). The GO analysis shows that in the DLPFC there was a functional enrichment of the selected genes in biological processes with crucial functions in neurotransmission, such as nucleotide binding (*NUBP1, PDXK*, and *STK32B*) and cell surface receptor-linked signal transduction (*GPR133* and *OR5A1*; *p* = 7.7e-3 for *GPR133*). The network analysis showed differently directed first-order protein interactions (Figure 2D); *PRKCE* was associated with genes that have different roles in the pathophysiology of the disease, such as *PTK2*, a key regulator of schizophrenia-related genes (Chandrasekaran and Bonchev, 2012), *ESR1*, an estrogen receptor that is altered in the frontal cortex of schizophrenic patients (Weickert et al., 2008), *YWHAZ*, which mediates signal transduction by binding to phosphoserine-containing proteins and has been associated with schizophrenia in a previous network analysis (Lee et al., 2011), and *TRPV1*, which plays a role in dopaminergic mechanisms associated with schizophrenia (Blumensohn et al., 2002). Finally, *CTNAP2* was associated with *CNTN2*, which is involved in the initial growth and guidance of axons and is downregulated in the superior temporal gyrus of schizophrenic patients (Roussos et al., 2012).

**Table 1:** **The 10 most differentially methylated probes after Bonferroni's correction (*p* < 10–7; β > 0.2) in the different areas analyzed between schizophrenic and healthy samples**.

	**Feature ID**	**Chr**	**Gene region**	**Schizophrenic**		**Healthy**		**Difference (mean)**
				**Mean**	***SD***	**Mean**	***SD***	
**DLPFC**								
	Undefined	4	Intergenic	0.65	0.13	0.06	0.04	0.59
	*NUBP1*	16	Body	0.89	0.03	0.46	0.00	0.43
	*STK32B*	4	Body	0.82	0.02	0.41	0.04	0.40
	*AIG1*	6	Body	0.81	0.03	0.42	0.02	0.38
	*PRKCE*	2	Body	0.81	0.02	0.43	0.00	0.38
	*FAM69C*	18	3′UTR	0.49	0.20	0.88	0.00	−0.38
	*RASA3*	13	Body	0.50	0.20	0.90	0.02	−0.40
	*RASA3*	13	Body	0.46	0.20	0.87	0.02	−0.41
	Undefined	17	Intergenic	0.50	0.17	0.93	0.04	−0.42
	*ATP11A*	13	3′UTR	0.33	0.14	0.90	0.01	−0.56
**HIPPOCAMPUS**								
	Non-defined	14	Intergenic	0.70	0.16	0.15	0.18	0.54
	*HLA-DQA1*	6	Body	0.60	0.13	0.10	0.07	0.49
	Non-defined	16	Intergenic	0.78	0.02	0.30	0.03	0.48
	*HCN2*	19	Body	0.59	0.14	0.13	0.12	0.46
	Non-defined	6	Intergenic	0.75	0.15	0.29	0.04	0.46
	Non-defined	16	Intergenic	0.87	0.13	0.42	0.10	0.44
	*AJAP1*	1	Body	0.16	0.19	0.55	0.20	−0.38
	*HLA-B*	6	3′UTR	0.14	0.05	0.57	0.20	−0.43
	Non-defined	2	Intergenic	0.38	0.01	0.87	0.00	−0.49
	*HLA-DRB5*	6	Body	0.36	0.19	0.88	0.05	−0.51
**ACC**								
	*C4orf50*	4	3′UTR	0.75	0.15	0.28	0.20	0.47
	Undefined	14	Intergenic	0.64	0.20	0.16	0.19	0.47
	*GALNT1*	18	5′UTR	0.84	0.03	0.45	0.06	0.39
	*VSX2*	14	TSS1500	0.54	0.18	0.16	0.20	0.38
	*SAPS2*	22	Body	0.80	0.09	0.44	0.08	0.35
	*KCNK7*	11	1st Exon	0.42	0.20	0.77	0.04	−0.35
	Undefined	14	Intergenic	0.35	0.18	0.71	0.04	−0.35
	*CSMD2*	1	Body	0.25	0.14	0.62	0.20	−0.37
	*FRK*	6	3′UTR	0.39	0.15	0.83	0.03	−0.43
	*TUBAL3*	10	TSS200	0.11	0.18	0.69	0.18	−0.58

Chr, Chromosome. TSS, Transcription start site.

**Figure 3:**
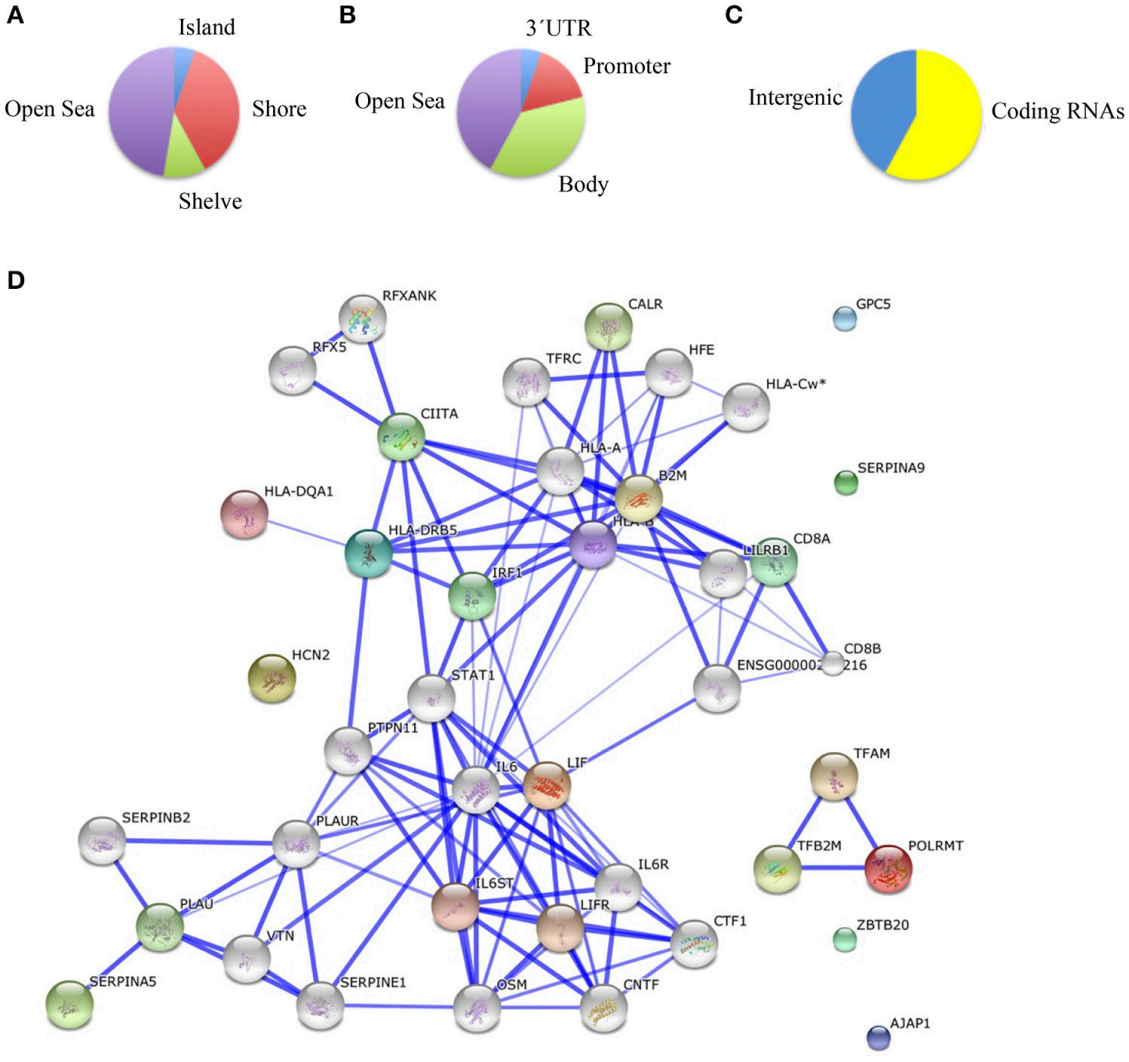
**(A)** Functional genomic distribution, **(B)** CpG content and neighborhood context, and **(C)** associated RNA transcripts of the 18 differentially methylated CpGs between schizophrenic and healthy samples in the hippocampus; **(D)** Network analysis (STRING V9.05).

Comparing methylation profiles revealed 8 hypermethylated and 10 hypomethylated CpGs in schizophrenic compared with healthy hippocampus, corresponding to 10 genes (Table 4S). In this case, most of the differentially methylated CpG sites were in CpG shores (36.8%) and CpGs isolated in the genome (47.3%; Figure 3A). From the functional genome distribution standpoint, most of the CpGs corresponded to gene body and intergenic-open sea sequences (47.3 and 38.8%, respectively; Figure 3B). With respect to their associated RNA transcripts, 57.8% were of classic coding messenger RNA genes, while for 42.1% sites there were no annotated transcripts associated with the described CpG location (Figure 3C). Again, we found more than 15% points in probes associated with gene body and coding RNA (Sandoval et al., 2011). The DAVID program identified one cluster, characterized by a highly significant enrichment of genes involved in antigen processing and presentation (*p* = 3.7e-5). In the known and predicted protein–protein interactions we draw attention to the associations of *HLA-DRB5* with *HLA-A* and *HLA-B* (Figure 3D), which are both involved in presenting foreign antigens to the immune system and are associated with schizophrenia (Chao et al., 2008; Childs et al., 2011; Gu et al., 2013), of *HLA-B* and *LIF* with *IL6* and *IL6R*, respectively, which have both been implicated in schizophrenia, although controversially in the case of the latter (Sun et al., 2008; Liu et al., 2010; Singh et al., 2011), of *HLB-B* with *HFE* and *CALR* (Farokhashtiani et al., 2011; Buretic-Tomljanovic et al., 2012) and, finally, of *LIF* with *LIFR*, which participates in signal transduction by members of the interleukin (IL)-6 cytokine family and has recently been associated with persecutory delusion in the Korean population (Kang et al., 2012).

**Figure 4:**
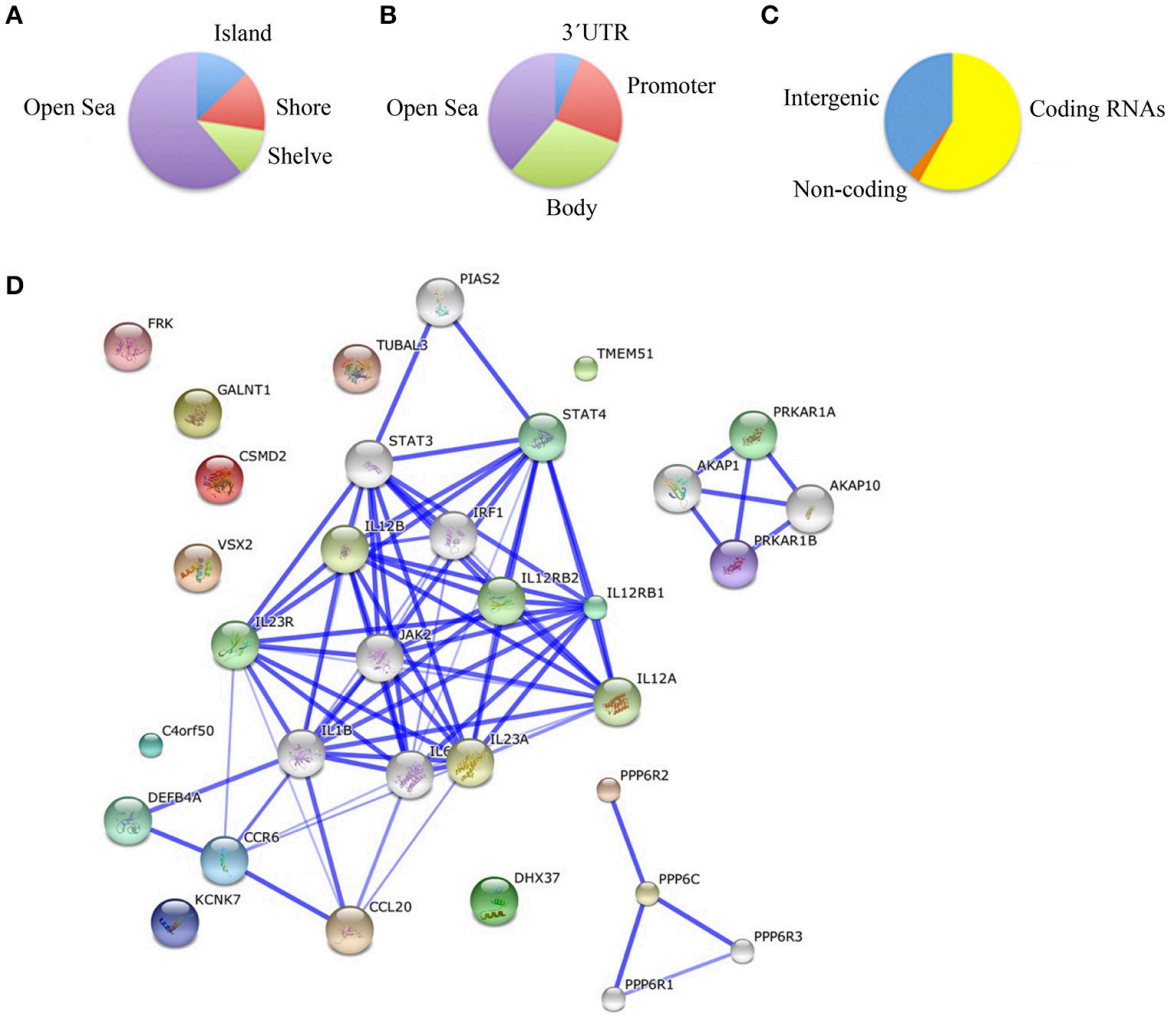
**(A)** Functional genomic distribution, **(B)** CpG content and neighborhood context, and **(C)** associated RNA transcripts of the 55 differentially methylated CpGs between schizophrenic and healthy samples in the ACC; **(D)** Network analysis in the ACC.

Finally, we compared schizophrenic ACC with corresponding healthy samples. The results are summarized in Table 5S and Figure 4. We found a total of 55 differentially methylated CpG sites corresponding to 32 genes, 41.9% hypermethylated (24/55), and 58.1% hypomethylated (31/55) in the schizophrenic ACC, recapitulating the global hypomethylation observed in this brain structure. As in the other brain structures analyzed, the distribution and functional location of the differentially methylated CpGs was variable (Figures 4A–C). In this brain area, we found a functional enrichment of the selected genes in biological processes such as protein kinase activity (*FRK, MAST2, KIAA1804*) and signal transduction (*SID1, IL12RB1, HLA-DRB5*). STRING analysis highlighted first-order protein interactions among *IL12RB1* and *IL12B, IL1B, IL6*, and *IL12A*, all of which have previously been associated with schizophrenia (Figure 4D) (Ozbey et al., 2008; Shirts et al., 2008; Fatjo-Vilas et al., 2012; Yoshida et al., 2012).

For probes indicated in Table 1 located at intergenic regions, we performed a transcription factor binding site prediction analysis and we did not find any known or predicted transcription factor that bind to these sequences. The results are summarized in Table 6S.

## Discussion

Herein, we compared the DNA methylation pattern across the human genome in several normal and schizophrenic brain areas that have previously been linked to neuropathological features of schizophrenia, such as DLPFC, hippocampus, and ACC. For our analysis we selected those patients with severe cognitive impairments and symptoms characteristic of the disease that are related with the aforementioned structures. Although, previous studies have analyzed the DNA methylation status of different gene regions in brain and peripheral blood samples (Mill et al., 2008; Dempster et al., 2011; Kinoshita et al., 2013; Nishioka et al., 2013; Alelu-Paz et al., 2015), to our knowledge, our study is the first high-resolution genome-wide DNA methylation analysis carried out in brain samples from patients with a severe cognitive impairment.

In this study, we analyzed a total of 485,476 cytosine positions distributed across the human genome, with an average of 17 CpG sites per gene region. We found that altered DNA methylation in schizophrenia is not restricted to the CpG island located at the promoter region, and includes others, such as CpG shelves and gene bodies. In our analysis, most of the CpG sites obtained were found to correspond to intergenic-open sea sequences. Epigenetic changes in these sequences (with previously unknown functions) have been shown to have crucial involvement in gene activation and de-activation (Hardison, 2012) and also, its methylation status might lead to facilitate or inhibit the binding of transcription factors (Jones, 2012). Therefore, our study furthers our understanding of the functional elements encoded in the human genome through an analysis not only of the gene regions that have traditionally been associated with gene regulation processes, but also of the intergenic regions that may potentially play a role in the pathophysiology of schizophrenia.

We know that methylation at CpG sites located in gene bodies is fundamental to the regulation of gene transcription elongation and could be also involved in the regulation of splicing (Jones, 2012). Although most of the gene bodies are poor in methylated CpGs, the intragenic regions also contain CpG islands (CGIs) that, with a few exceptions, are unmethylated. This is particularly important since as many as 34% of these CGIs are methylated in the human brain, suggesting a tissue-specific role for this methylation (Maunakea et al., 2010). Thus, the changes in the methylation state of the probes located in intragenic regions observed here could be highly significant for the development of schizophrenia. In this regard, we found that *LIF* (leukemia inhibitory factor), which is hypomethylated in the hippocampus of samples from schizophrenic patients, could have an important role in the cognitive impairment associated with schizophrenic disabilities, since it induces neuronal cell differentiation and participates in the deterioration of working memory function (Okahisa et al., 2010). *PRKCE* and *CNTNAP2* are two other genes that are hypermethylated in the DLPFC of schizophrenic patients. Both are thought to be potential contributors to the central nervous system pathology in schizophrenia (Nawa et al., 2000; Roussos et al., 2012). *PRKCE* is involved in nerve growth factor (NFG)-induced neurite outgrowth and neuron morphological change. *CNTNAP2* may play a major role in the formation of functionally distinct domains critical for saltatory conduction of nerve impulses in myelinated nerve fibers and, therefore, its epigenetic alteration is consistent with the hypothesis that schizophrenia results from poor or miswired anatomical or functional connections (Salgado-Pineda et al., 2007).

In psychiatry, most of the studies have tried to elucidate the role of DNA methylation changes by using peripheral blood samples. This choice is usually justified by the mounting evidence from other disorders in which disease-associated epimutations can be detected across different tissue types (Dempster et al., 2011; Kinoshita et al., 2013). However, DNA methylation profile is tissue-specific, and the epigenetic signatures are not always correlated with those obtained from DNA isolated from peripheral blood samples (Ladd-Acosta et al., 2007; Davies et al., 2012). Taking this fact into account, we compared our results with the previous methylation study carried out in peripheral blood samples from schizophrenic patients (Kinoshita et al., 2013) being unable to replicate the DNA methylation changes they observed in that tissue. By using brain samples, instead leukocytes, we found a global hypermethylation pattern in all the structures analyzed in schizophrenic and healthy individuals in contrast to previous findings from peripheral blood samples that indicated highly significant hypomethylation in patients with schizophrenia (Shimabukuro et al., 2007; Melas et al., 2012). This observation highlights the idea that the epigenetic signatures differ depending on whether brain or blood samples are analyzed.

Even more, these signatures differ between brain regions. In this sense, in our study, only nine differentially methylated probes (Δβ > 0.2; *p* < 0.01, *SD* < 20%) were present in at least two of the three areas included, corresponding to intergenic regions (six probes) and three corresponding to genes (*ATP2A3*-Body, *AIG1*-Body, *HLA-DRB5*-Body). No probe was repeated across all three brain areas analyzed, so we can conclude that the schizophrenic and normal human brain have different anatomical DNA methylation signatures, which are brain-region dependent.

Therefore, we wished to highlight the need to study brain samples to obtain reliable data in regard to the epigenetic study of this severe mental illness. The more we understand the dynamic nature of epigenetic the more we need to take care of the wise tissue selection and its adequate anatomical identification. The conjunction of these factors will allow us to deepen our knowledge in schizophrenia, avoiding data that add “noise” instead truly information, and leading to a more exact diagnose, treatment, and patients' life-quality improvement.

## Author contributions

RA, FC, JVS, AG, NA, MO, AE, AM, CG, AC, JS, ME, SR: acquired, analyzed, and interpreted data for the work. This also included, drafting the work or revising it critically for important intellectual content. RA, FC, JVS, AG, NA, MO, AE, AM, CG, AC, JS, ME, SR: Agreement to be accountable for all aspects of the work in ensuring that questions related to the accuracy or integrity of any part of the work are appropriately investigated and resolved. RA, FC, JVS, AG, NA, MO, AE, AM, CG, AC, JS, ME, SR: Final approval of the version to be submitted.

### Conflict of interest statement

The authors declare that the research was conducted in the absence of any commercial or financial relationships that could be construed as a potential conflict of interest.
