# Supplementary material for: Epigenetics in Schizophrenia: A Pilot Study of Global DNA Methylation in Different Brain Regions Associated with Higher Cognitive Functions
Source: Front Psychol. 2016 Sep 30;7:1496. doi: 10.3389/fpsyg.2016.01496 (PMC5044511; doi:10.3389/fpsyg.2016.01496)
Supplement: Supplementary file 1 [file DataSheet1.docx]

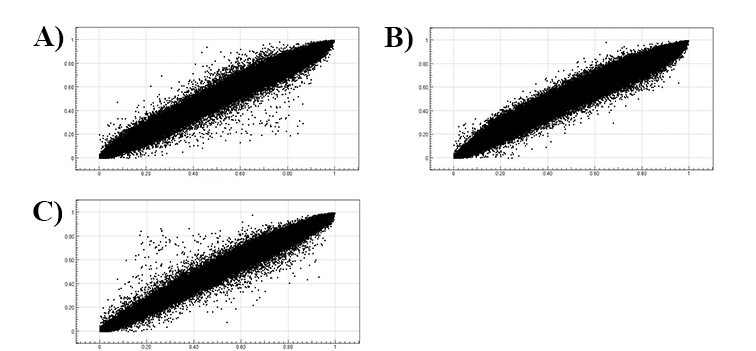


*FIGURE 1S. Scatter plot for CpG methylation values (AVGβ) among the brain structures included in our analysis. Healthy samples. A) DLPFC vs Hippocampus (Pearson correlation coefficient R^2^= 0.9935); B) DLPC vs ACC (Pearson correlation coefficient R^2^= 0.9918) and C) Hippocampus vs ACC (Pearson correlation coefficient R^2^= 0.9950)*


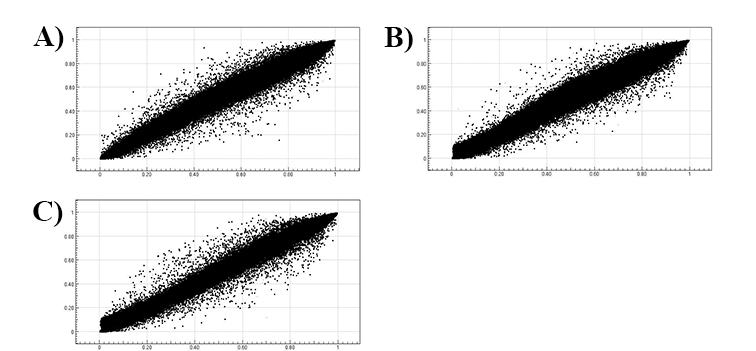


*FIGURE 2S. Scatter plot for CpG methylation values (AVGβ) among the brain structures included in our analysis. Schizophrenic samples. A) DLPFC vs Hippocampus (Pearson correlation coefficient R^2^= 0.9944); B) DLPFC vs ACC (Pearson correlation coefficient R^2^= 0.9932) and C) Hippocampus vs ACC (Pearson correlation coefficient R^2^= 0.9963).*
