# Supplementary material for: Epigenetics in Schizophrenia: A Pilot Study of Global DNA Methylation in Different Brain Regions Associated with Higher Cognitive Functions
Source: Front Psychol. 2016 Sep 30;7:1496. doi: 10.3389/fpsyg.2016.01496 (PMC5044511; doi:10.3389/fpsyg.2016.01496)
Supplement: Supplementary file 2 [file DataSheet2.docx]

| **Variable** | **Schizophrenia** | **Controls** |
| --- | --- | --- |
| Number of subjects | 19 | 3 |
| Gender (Male-Female) | 19M | 3M |
| Age at death | 80.72 ± 9.46 | 75± 8.66 |
| Post-mortem interval (hours) | 3.94 ± 2.20 | 11± 7.81 |
| Psychiatric diagnosis | Schizophrenia. Residual Type (295.60) | None |

*Table 1S. Demographic details of samples included in the study*

|  | **Healthy samples** | | **Schizophrenic samples** | |
| --- | --- | --- | --- | --- |
|  | Probes hypermethylated | Probes hypomethylated | Probes hypermethylated | Probes hypomethylated |
|  |  |  |  |  |
| **DLPFC** | 195765 | 160654 | 194385 | 160914 |
| **Hippocampus** | 196537 | 162531 | 194848 | 162535 |
| **ACC** | 192782 | 164039 | 193371 | 161460 |

*Table 2S. Total number of hyper and hypomethylated probes both in schizophrenic and healthy samples.*

|  | | | **Schizophrenia** | | **Healthy** | |  |
| --- | --- | --- | --- | --- | --- | --- | --- |
| **Feature ID** | **Chr** | **Gene region** | **Mean** | **SD** | **Mean** | **SD** | \| **Difference (means)** \| \| --- \| |
| Non defined | 4 | Intergenic | .65 | .13 | .06 | .04 | .59 |
| *NUBP1* | 16 | Body | .89 | .03 | .46 | .00 | .43 |
| *RASA3* | 13 | Body | .46 | .20 | .87 | .02 | .41 |
| *STK32B* | 4 | Body | .82 | .02 | .41 | .04 | .40 |
| *AIG1* | 6 | Body | .81 | .03 | .42 | .02 | .38 |
| *PRKCE* | 2 | Body | .81 | .02 | .43 | .00 | .38 |
| *CNTNAP2* | 7 | Body | .77 | .17 | .39 | .02 | .37 |
| *AHSP* | 16 | TSS1500 | .75 | .17 | .38 | .01 | .37 |
| Non defined | 5 | Intergenic | .83 | .18 | .46 | .02 | .36 |
| *C22orf43* | 22 | TSS1500 | .80 | .15 | .44 | .02 | .36 |
| Non defined | 2 | Intergenic | .81 | .13 | .47 | .02 | .34 |
| *ATP2A3* | 17 | Body | .68 | .15 | .34 | .02 | .33 |
| Non defined | 15 | Intergenic | .36 | .16 | .02 | .00 | .33 |
| *DENND2A* | 7 | Body | .42 | .10 | .13 | .14 | .30 |
| *OR5A1* | 11 | TSS1500 | .42 | .16 | .14 | .03 | .29 |
| *FOLH1B* | 11 | TSS1500 | .68 | .17 | .41 | .05 | .28 |
| *RPL28* | 19 | TSS1500 | .32 | .19 | .05 | .08 | .28 |
| *GPR133* | 12 | Body | .34 | .06 | .08 | .07 | .27 |
| *AMN* | 14 | TSS200 | .45 | .07 | .20 | .10 | .26 |
| Non defined | 6 | Intergenic | .40 | .08 | .15 | .11 | .26 |
| *MUC5B* | 11 | Body | .61 | .20 | .36 | .03 | .25 |
| *DOCK1* | 10 | Body | .60 | .14 | .36 | .02 | .25 |
| Non defined | 6 | Intergenic | .61 | .18 | .37 | .00 | .24 |
| *KIF26A* | 14 | Body | .53 | .14 | .29 | .06 | .24 |
| *ARSB* | 5 | TSS1500 | .26 | .20 | .03 | .01 | .24 |
| *KBTBD11* | 8 | 5'UTR | .71 | .16 | .49 | .04 | .23 |
| *GFPT2* | 5 | Body | .68 | .18 | .46 | .04 | .23 |
| Non defined | 14 | Intergenic | .58 | .14 | .36 | .06 | .23 |
| Non defined | 1 | Intergenic | .53 | .09 | .31 | .02 | .22 |
| *GREB1* | 2 | TSS1500 | .26 | .17 | .05 | .01 | .22 |
| Non defined | 14 | Intergenic | .51 | .14 | .30 | .04 | .21 |
| Non defined | 1 | Intergenic | .48 | .17 | .27 | .03 | .21 |
| Non defined | 1 | Intergenic | .25 | .14 | .05 | .02 | .21 |
| Non defined | 9 | Intergenic | .48 | .16 | .28 | .01 | .20 |
| Non defined | 9 | Intergenic | .46 | .14 | .26 | .02 | .20 |
| Non defined | 18 | Intergenic | .65 | .14 | .86 | .03 | -.20 |
| *LEPREL1* | 3 | 5'UTR | .57 | .17 | .78 | .04 | -.20 |
| Non defined | 4 | Intergenic | .36 | .16 | .58 | .02 | -.21 |
| *PDXK* | 21 | Body | .25 | .07 | .47 | .05 | -.21 |
| Non defined | 18 | Intergenic | .40 | .09 | .63 | .03 | -.22 |
| *ZBTB46* | 20 | Body | .39 | .13 | .62 | .03 | -.22 |
| *PKP3* | 11 | Body | .69 | .17 | .93 | .01 | -.23 |
| Non defined | 8 | Intergenic | .57 | .15 | .81 | .02 | -.23 |
| Non defined | 10 | Intergenic | .14 | .17 | .38 | .03 | -.23 |
| *SNTB1* | 8 | 1^st^Exon | .13 | .14 | .37 | .04 | -.23 |
| Non defined | 16 | Intergenic | .67 | .16 | .92 | .01 | -.24 |
| *NTRK3* | 15 | Body | .49 | .12 | .73 | .08 | -.24 |
| *NID2* | 14 | Body | .27 | .14 | .52 | .03 | -.24 |
| Non defined | 1 | Intergenic | .60 | .18 | .86 | .01 | -.25 |
| *POTEH* | 22 | TSS1500 | .58 | .06 | .86 | .03 | -.27 |
| Non defined | 7 | Intergenic | .54 | .20 | .82 | .02 | -.27 |
| Non defined | 8 | Intergenic | .44 | .20 | .72 | .09 | -.27 |
| *KCNJ5* | 11 | 3'UTR | .13 | .11 | .41 | .06 | -.27 |
| *NR1H4* | 12 | Body | .62 | .20 | .91 | .01 | -.29 |
| Non defined | 4 | Intergenic | .15 | .12 | .44 | .12 | -.29 |
| *HLA-C* | 6 | Body | .14 | .17 | .44 | .08 | -.30 |
| Non defined | 4 | Intergenic | .44 | .18 | .77 | .06 | -.32 |
| Non defined | 12 | Intergenic | .54 | .16 | .87 | .02 | -.33 |
| *SSBP3* | 1 | Body | .45 | .15 | .79 | .02 | -.33 |
| Non defined | 1 | Intergenic | .36 | .20 | .70 | .03 | -.34 |
| Non defined | 1 | Intergenic | .50 | .16 | .86 | .01 | -.35 |
| *RASA3* | 13 | Body | .44 | .17 | .82 | .00 | -.37 |
| *FAM69C* | 18 | 3'UTR | .49 | .20 | .88 | .00 | -.38 |
| *RASA3* | 13 | Body | .50 | .20 | .90 | .02 | -.40 |
| Non defined | 17 | Intergenic | .50 | .17 | .93 | .04 | -.42 |
| *ATP11A* | 13 | 3'UTR | .33 | .14 | .90 | .01 | -.56 |

*Table 3S. Differentially methylated probes after Bonferroni´s correction (P < 10^-7^;* *β > 0.2) in the DLPFC between schizophrenic and healthy samples. Chr, Chromosome. TSS, Transcription Start Site*

|  | | | **Schizophrenia** | | **Healthy** | |  |
| --- | --- | --- | --- | --- | --- | --- | --- |
| **Feature ID** | **Chr** | **Gene region** | **Mean** | **SD** | **Mean** | **SD** | \| **Difference (means)** \| \| --- \| |
| Non defined | 14 | Intergenic | .70 | .16 | .15 | .18 | .54 |
| *HLA-DQA1* | 6 | Body | .60 | .13 | .10 | .07 | .49 |
| Non defined | 16 | Intergenic | .78 | .02 | .30 | .03 | .48 |
| *HCN2* | 19 | Body | .59 | .14 | .13 | .12 | .46 |
| Non defined | 6 | Intergenic | .75 | .15 | .29 | .04 | .46 |
| Non defined | 16 | Intergenic | .87 | .13 | .42 | .10 | .44 |
| *GPC5* | 13 | Body | .49 | .12 | .14 | .13 | .34 |
| Non defined | 4 | Intergenic | .58 | .10 | .24 | .18 | .33 |
| *SERPINA5* | 14 | TSS1500 | .55 | .17 | .93 | .00 | -.37 |
| *POLRMT* | 19 | Body | .51 | .18 | .88 | .04 | -.37 |
| *SERPINA9* | 14 | TSS1500 | .47 | .19 | .85 | .00 | -.37 |
| Non defined | 14 | Intergenic | .37 | .15 | .74 | .02 | -.37 |
| Non defined | 2 | Intergenic | .30 | .10 | .68 | .18 | -.37 |
| *LIF* | 22 | Body | .50 | .17 | .88 | .00 | -.38 |
| *AJAP1* | 1 | Body | .16 | .19 | .55 | .20 | -.38 |
| *HLA-B* | 6 | 3'UTR | .14 | .05 | .57 | .20 | -.43 |
| Non defined | 2 | Intergenic | .38 | .01 | .87 | .00 | -.49 |
| *HLA-DRB5* | 6 | Body | .36 | .19 | .88 | .05 | -.51 |

*Table 4S. Differentially methylated probes after Bonferroni´s correction (P < 10^-7^;* *β > 0.2) in the hippocampus between schizophrenic and healthy samples. Chr, Chromosome. TSS, Transcription Start Site*

|  | | | **Schizophrenia** | | **Healthy** | |  |
| --- | --- | --- | --- | --- | --- | --- | --- |
| **Feature ID** | **Chr** | **Gene region** | **Mean** | **SD** | **Mean** | **SD** | \| **Difference (means)** \| \| --- \| |
| *C4orf50* | 4 | 3'UTR | .75 | .15 | .28 | .20 | .47 |
| Non defined | 14 | Intergenic | .64 | .20 | .16 | .19 | .47 |
| *GALNT1* | 18 | 5'UTR | .84 | .03 | .45 | .06 | .39 |
| *VSX2* | 14 | TSS1500 | .54 | .18 | .16 | .20 | .38 |
| *SAPS2* | 22 | Body | .80 | .09 | .44 | .08 | .35 |
| Non defined | 22 | Intergenic | .76 | .16 | .45 | .03 | .30 |
| *TMEM51* | 1 | 5'UTR | .63 | .12 | .33 | .05 | .29 |
| Non defined | 14 | Intergenic | .62 | .18 | .32 | .04 | .29 |
| *SIDT1* | 3 | Body | .80 | .07 | .51 | .16 | .28 |
| *COL4A2* | 13 | Body | .71 | .19 | .44 | .01 | .28 |
| *DHX37* | 12 | Body | .69 | .19 | .40 | .03 | .28 |
| Non defined | 14 | Intergenic | .46 | .10 | .19 | .08 | .27 |
| Non defined | 14 | Intergenic | .31 | .19 | .04 | .00 | .27 |
| *KIAA0753* | 17 | 5'UTR | .71 | .13 | .45 | .04 | .26 |
| *RPTOR* | 17 | Body | .59 | .11 | .33 | .03 | .26 |
| *ATP2A3* | 17 | Body | .64 | .17 | .39 | .04 | .25 |
| Non defined | 13 | Intergenic | .85 | .05 | .62 | .04 | .23 |
| *AIG1* | 6 | Body | .65 | .20 | .43 | .01 | .23 |
| *PCDHB13* | 5 | 1^st^Exon | .52 | .16 | .29 | .04 | .23 |
| *MYT1L* | 2 | Body | .73 | .17 | .51 | .05 | .22 |
| *CST9L* | 20 | TSS1500 | .72 | .20 | .50 | .03 | .22 |
| Non defined | 6 | Intergenic | .33 | .15 | .11 | .08 | .22 |
| *MAST2* | 1 | Body | .59 | .18 | .38 | .04 | .21 |
| *ELN* | 7 | 1^st^Exon | .25 | .13 | .05 | .02 | .20 |
| *KIAA1530* | 4 | Body | .74 | .14 | .94 | .01 | -.20 |
| *LOC728613* | 5 | Body | .61 | .18 | .81 | .01 | -.20 |
| *HLA-DRB6* | 6 | Body | .54 | .15 | .74 | .06 | -.20 |
| *GPR20* | 8 | 5'UTR | .43 | .13 | .63 | .02 | -.20 |
| *CLPTM1L* | 5 | Body | .63 | .18 | .84 | .03 | -.21 |
| *DLC1* | 8 | Body | .60 | .16 | .81 | .02 | -.21 |
| Non defined | 3 | Intergenic | .58 | .17 | .80 | .01 | -.21 |
| *WDR66* | 12 | TSS1500 | .36 | .05 | .57 | .12 | -.21 |
| Non defined | 4 | Intergenic | .13 | .08 | .34 | .07 | -.21 |
| *MORF4* | 4 | TSS1500 | .65 | .20 | .87 | .02 | -.22 |
| Non defined | 8 | Intergenic | .59 | .11 | .80 | .05 | -.22 |
| Non defined | 1 | Intergenic | .55 | .18 | .77 | .03 | -.22 |
| Non defined | 8 | Intergenic | .51 | .10 | .72 | .10 | -.22 |
| Non defined | 4 | Intergenic | .14 | .12 | .36 | .05 | -.22 |
| Non defined | 3 | Intergenic | .70 | .20 | .93 | .02 | -.23 |
| Non defined | 1 | Intergenic | .47 | .16 | .70 | .01 | -.23 |
| Non defined | 4 | Intergenic | .36 | .20 | .61 | .04 | -.25 |
| *IRS2* | 13 | Body | .33 | .18 | .58 | .02 | -.25 |
| *HLA-DRB5* | 6 | Body | .30 | .13 | .55 | .13 | -.25 |
| Non defined | 4 | Intergenic | .18 | .12 | .44 | .07 | -.26 |
| Non defined | 16 | Intergenic | .48 | .18 | .77 | .02 | -.29 |
| Non defined | 10 | Intergenic | .54 | .14 | .84 | .02 | -.30 |
| *IL12RB1* | 19 | 3'UTR | .45 | .14 | .76 | .02 | -.30 |
| *PRKAR1B* | 7 | Body | .27 | .20 | .59 | .04 | -.31 |
| Non defined | 15 | Intergenic | .49 | .13 | .82 | .02 | -.32 |
| Non defined | 15 | Intergenic | .16 | .15 | .48 | .03 | -.32 |
| *KCNK7* | 11 | 1^st^Exon | .42 | .20 | .77 | .04 | -.35 |
| Non defined | 14 | Intergenic | .35 | .18 | .71 | .04 | -.35 |
| *CSMD2* | 1 | Body | .25 | .14 | .62 | .20 | -.37 |
| *FRK* | 6 | 3'UTR | .39 | .15 | .83 | .03 | -.43 |
| *TUBAL3* | 10 | TSS200 | .11 | .18 | .69 | .18 | -.58 |

*Table 5S. Differentially methylated probes after Bonferroni´s correction (P < 10^-7^;* *β > 0.2) in the ACC between schizophrenic and healthy samples. Chr, Chromosome. TSS, Transcription Start Site*

| **Chromosome** | **Sequence** | **Known/Predicted Transcription Factor Binding Site**  *(UCSC Genome Browser GRCh37/hg19)* |
| --- | --- | --- |
|  |  |  |
| **DLPFC** | | |
| 4 | CGGCCAATGTCTAAGCCTAGACCATCTGTGGGCAGTTATTTCAAAGAATC | None |
| 17 | TCAGGAGGATAATTCTGAGCCAGGGGTGGGGGTACCGGCCTGGAGCTCCG | None |
| **Hippocampus** | | |
| 14 | TGAGAGCAGAAGAAAAAATTAACCTCACACACTTACCTCAAATTGTTTGCCCATCTAGTTCGTTTGTTGCCAGAAACTTAGATTCAGTAGACTCAACAGCACATGAAGCAAGAATTCCCGAA | None |
| 16 | CAGTGACTCCAGACATCACACACGACACTGTGGTCACCGGGGAAAATGGGCTGTTTCTCCCGAGGCATCTCTTGTTTTGTGGTGAAACAAACCTTCTTGGATTTGTCCTCACCTTCATGGGC | None |
| 6 | AAGAAGTGCCACAATATGCCATCTGCAAGTAGGAGACCCAGGAAAGCAGGTGGTGTAATTCGGTGAGAGTCAGAAGGGTTGAGGATCAAGGGAGCCAATGGTACAACTCCCAGTCTGAGGCC | None |
| 16 | GGGGCACAGACGTGGTCAGAGGGGCACACGGGTAATGCACGGATGTGGTCGGGGGGCACACGGGTGAGGCGTGGACATGGTCAGTGGACGGGGCTTGGCGTGAGCTCCTGGAGTTAGAGGGC | None |
| 2 | ACAGGCAGGAGCCGGGACAGGCAGGACCCGGGACAGGCAGGACCCGGGAAAGGCAGGACCCGGGACAGGCAGGACCTGGGACAGGCAGCCATTGCTCTGGAACCCTCCCAGTGTGGATAGAG | None |
| **ACC** | | |
| 14 | CCTGGTGGAAAAATCCAGAAGACCCTCTCCCTGAGCATGAGTGGGGTGGTCAGAGGCCTCCGGGTGAGGAGACAGATGGGGCCTGCCTTGCTGCCCTGGGCTGGGGCTGCACAGCCGGGGTG | None |
| 14 | CGGGAGGGGACCCGCGGCTGAGGGGACAGGTCCTGCGTGCGGGCTGGGGAGACAGGCGGGCGAGGGTAGGGGTGCACAAGGCTGTGTGGTTTGTGGGCTGTTGTGCAGGGTGGGTCCAAGCA | None |

*Table 6S. Transcription binding sites prediction study for the most differentially methylated probes (p<0.01;* *β>0.2) located at intergenic regions from Table 1. DLPFC: dorsolateral prefrontal cortex. ACC: anterior cingulate cortex.*
